# Indium-Mediated Allylation of Carbonyl Compounds in Ionic Liquids: Effect of Salts in Ionic Liquids

**DOI:** 10.3390/molecules23071696

**Published:** 2018-07-11

**Authors:** Tsunehisa Hirashita, Fusako Takahashi, Takayuki Noda, Yuji Takagi, Shuki Araki

**Affiliations:** Life Science and Applied Chemistry, Graduate School of Engineering, Nagoya Institute of Technology, Gokiso-cho, Showa-ku, Nagoya 466-8555, Japan; f.takahashi.144@nitech.jp (F.T.); t.noda.686@nitech.jp (T.N.); y.takagi.570@nitech.jp (Y.T.); a510224@hotmail.co.jp (S.A.)

**Keywords:** indium, allylation, ionic liquids, carbonyl compounds, homoallylic alcohols

## Abstract

The In-mediated allylation of carbonyl compounds can be performed in various types of solvents including ionic liquids. However, we have found that in [bmim][BF_4_] (where bmim = 1-butyl-3-methylimidazolium), the In-mediated coupling of crotyl bromide with benzaldehyde gives a complex mixture, and some additives, such as halides and amines, are crucial for the successful conversion to the corresponding γ-adduct. Instead, the addition of alcohols or water promotes the formation of the α-adduct. An asymmetric induction with up to 62% enantiomeric excess (ee) was observed employing cinchonidine as an additive in a binary solvent consisting of an ionic liquid and dichloromethane.

## 1. Introduction

Indium-mediated allylations of carbonyl compounds have been extensively studied during the last two decades [1,2,3,4]. Allylindium reagents are prepared from metallic indium and allylic halides in a broad range of solvents, including water, and can be used in the Barbier-type allylation of carbonyl compounds. We previously reported the allylation of carbonyl compounds with catalytic amounts of indium(III) trichloride and stoichiometric amounts of aluminum (0) in aqueous tetrahydrofuran [5]. This method provides an easy access to reactions of allylic indium reagents and employs reduced quantities of the relatively expensive indium metal. However, this catalytic allylation proceeds slowly and a couple of days are needed to complete the reaction. More recently, we reported a catalytic variation in a binary solvent system constituted of water and an ionic liquid [6].

Ionic liquids have emerged as recyclable solvents in organic synthesis, and their electrochemical properties have also received attention. The preparation of organometallic compounds in ionic liquids has also been of interest. Grignard reagents, however, have not been prepared in ordinary ionic liquids, and a chelating group such as an ether functionality is required in the ionic liquids to stabilize the Grignard reagents [7,8]. The preparation of allylic indium reagents and their reactions with aldehydes [9,10,11,12] or imines [13] in ionic liquids have been reported, where the corresponding homoallylic alcohols or homoallylic amines have been obtained in high yields. Noteworthy, the use of ionic liquids possessing chelating units is not required for promoting allylation when allylic indium reagents are involved. Herein, we report that, in contrast to previous results, there are limitations in using ionic liquids for preparing allylic indium reagents, and special additives, including halides, are required in the crotylation of aldehydes depending on the nature of the ionic liquid employed.

## 2. Results and Discussion

### 2.1. Allylation

We began screening a wide range of ionic liquids to perform the Barbier-type In-mediated allylation. Thus, when allyl bromide and benzaldehyde were mixed with indium powder in [bmim][BF_4_] (where bmim = 1-butyl-3-methylimidazolium), the corresponding homoallylic alcohol **1** was obtained in good yield and in a shorter time (5 h) than that previously reported [10] (entry 1, Table 1). The reactions in [bpy][BF_4_] (where bpy = *N*-butylpyridinium) and [bmim][(CN)_2_N] also gave **1** in high yields (entries 2 and 3), while the yield decreased in [bmim][OTf] (entry 4). The reaction in [bmim][Cl], which was performed under conventional heating as [bmim][Cl] is not liquid at room temperature, gave **1** in low yield (entry 5). The allylation in [bmim][NTf_2_] (where NTf_2_ = bis(trifluoromethanesulfonyl)amide) did not proceed (entry 6).

We next focused on the allylation involving a reductive transmetalation of a π-allylpalladium(II) with In(I), in which a variety of solvents can be used [14]. Among the ionic liquids employed, as above [bmim][BF_4_] gave the best yield (entry 1, Table 2). The reactions in [bpy][BF_4_] and [bmim][OTf] proceeded in moderate yields (entries 2 and 3), while [bmim][(CN)_2_N] was not suitable for the allylation (entry 4).

### 2.2. Crotylation

**Figure 1:**
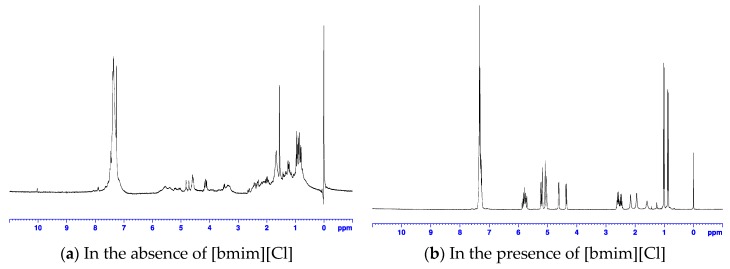
Crotylation of benzaldehyde in [bmim][BF_4_] in the absence (**a**) or presence (**b**) of [bmim][Cl].

We then turned our attention to the crotylation of benzaldehyde. The conversions in [bmim][(CN)_2_N] and [bmim][OTf] proceeded well affording homoallylic alcohol **2** in good yields (entries 1 and 2, Table 3). Instead, [bmim][BF_4_], which had proved advantageous in the above reactions (Table 1 and Table 2), gave a complicated mixture when used for the coupling of crotyl bromide and benzaldehyde. The ^1^H-NMR spectrum of the crude mixture is shown in Figure 1a. As the crotylation in [bmim][BF_4_] had previously been successful [10], the above result suggests that there are problems associated with the source of the ionic liquid employed. We anticipated that the difference may be caused by some type of impurity present in the ionic liquid, such as [bmim][X] the precursor of [bmim][BF_4_], which presumably plays an essential role in the allylation of aldehydes as it does in the allylation of imines [13].

**Scheme 1:**
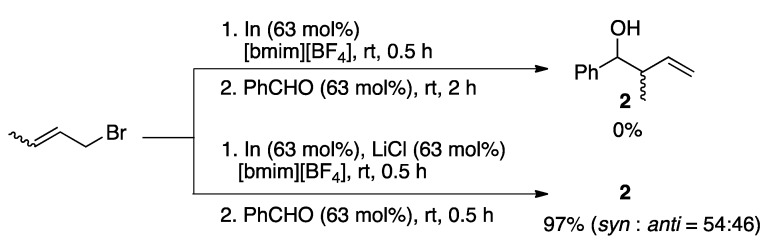
Effects of LiCl on the crotylation of benzaldehyde.

We then tested whether the [bmim][Cl] residue in [bmim][BF_4_] affected the crotylation. When crotyl bromide and benzaldehyde were stirred with indium powder in [bmim][BF_4_] containing a small amount of [bmim][Cl], homoallylic alcohol **2** was formed cleanly as depicted in Figure 1b (entry 1, Table 4). In place of [bmim][Cl], LiCl was next examined to clarify the role of halides. The addition of a stoichiometric amount of LiCl also improved the yield, giving **2** in high yield (entry 2). The reactions with either a catalytic or excess amount of LiCl gave lower yields (entries 3 and 4). To get further insights into the role of LiCl in the mechanism of the reaction, we carried out a set of Grignard-type reactions; thus, a mixture of crotyl bromide and indium in [bmim][BF_4_] was stirred at room temperature for 0.5 h in the presence or absence of LiCl prior to the addition of benzaldehyde. Without LiCl, the reaction did not give homoallylic alcohol **2**. Instead, the reaction with LiCl afforded **2** in 97% yield (Scheme 1).

During the reaction of crotyl bromide with indium in [bmim][BF_4_] in the absence of LiCl, the consumption of indium powder was sluggish and an unidentified solid was deposited [15]. These results demonstrate that LiCl stabilizes both the crotylindium reagent and the resulting indium homoallylic alkoxide and prevents from unexpected reactions leading to a decreased yield of **2**. The addition of catalytic amounts (10 mol %) of [bmim][Cl], [bmim][Br], or LiBr was sufficient for a clean formation of **2** (entries 5–7), which probably relates to the tendency of these halides to dissociate and their solubility in the media. We next set out to examine other organic/inorganic salts in the crotylation. The addition of triphenylphosphine gave a good yield (entry 8). While a primary alkylamine worked as a promoter to some extent, tertiary amines were less efficient (entries 9 and 10). Pyridine and cinchonidine [16] proved to be good promoters (entries 11 and 12) [17]. Some alcohols, except phenol, promoted the crotylation. However, linear homoallylic alcohol **3** was formed as the major product (entries 13–15). The addition of water also gave a mixture of **2** and **3** (entry 16), indicating that an oxonia[3,3]-sigmatropic rearrangement occurs under these conditions [18].

### 2.3. Intramolecular Auxiliary

**Scheme 2:**
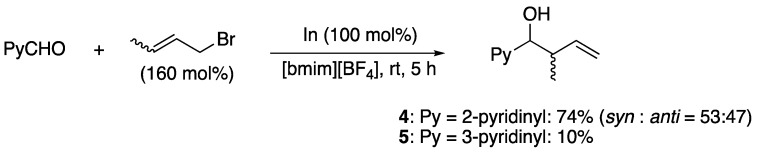
Effects of intramolecular chelating units.

As pyridine proved to function as a good promoter, we next employed 2- and 3-pyridinecarboxaldehydes instead of benzaldehyde as substrates of the crotylation reaction in the absence of additives in order to assess the influence of intramolecular interactions between the pyridyl and carbonyl groups (Scheme 2). Although both aldehydes gave the corresponding homoallylic alcohols **4** and **5**, 2-pyridinecarboxaldehyde gave a higher yield of the corresponding product than the reaction performed with the 3-pyridinyl isomer. This result suggests that the ring nitrogen atom adjacent to the carbonyl group not only stabilizes the crotylindium reagent and derivatives thereof in [bmim][BF_4_] but also helps the rapid transformation by intramolecular chelation [19,20].

### 2.4. Chiral Auxiliary

The importance of intra- and intermolecular chelating groups to promote a clean crotylation prompted us to examine an asymmetric version of the present reaction. We anticipated that an asymmetric induction would function well with a judicious choice of chiral additives due to their critical role in promoting the allylation in [bmim][BF_4_]. As the use of cinchonidine had been reported by Loh and co-workers as a suitable chiral auxiliary for the asymmetric addition of allylindium reagents to aldehydes [21], we set to investigate the effect of cinchonidine on the allylation of benzaldehyde in [bmim][BF_4_] under similar conditions to those reported by Loh. After stirring a mixture of allyl bromide and cinchonidine with indium in [bmim][BF_4_] for 3 h at room temperature, benzaldehyde was added. The reaction in [bmim][BF_4_] gave a lower yield than that performed without additive (entry 1, Table 1), and a neglectable ee was observed (entry 1, Table 5). We then examined the reaction at low temperature, where the ionic liquid needed to be diluted with an organic solvent due to its high viscosity, and thus, CH_2_Cl_2_ was chosen due to its weakly coordinating character. The reaction in the binary solvent gave a good yield at room temperature with a moderate ee (entry 2), while at −78 °C both the yield and ee remarkably decreased (entry 3). At 40 °C, a somewhat higher ee was observed (entry 4). Because cinchonidine is poorly soluble in [bmim][BF_4_], its benzyl ammonium salt was next employed in the reaction (entry 5). This salt completely dissolved and the reaction quickly proceeded, albeit the ee significantly decreased. The reaction in [bpy][BF_4_] showed a similar tendency to that found in [bmim][BF_4_]; the ee value decreased at low temperature (entries 6 and 7). The reaction in a mixture of [bmim][OTf] and CH_2_Cl_2_ also perceived an improvement both in yield and ee, while the opposite phenomena to that found in [bmim][BF_4_] was observed at low temperature, and the ee increased as a result of lowering the temperature (entries 8–11).

## 3. Materials and Methods

### 3.1. Analysis

^1^H-NMR spectra were recorded on a Varian Mercury spectrometer (300 MHz). Please see the ^1^H-NMR of compounds **1**–**5** in Supplementary Materials. The optical purities of the products were measured by HPLC (Daicel Chiralcel OD-H column). 1-Phenyl-3-buten-1-ol: hexane/*i*-PrOH = 98:2, flow rate 1.0 mL min^−1^, t_R_ = 17.5 min (*R*)-enantiomer, t_R_ = 20.9 min (*S*)-enantiomer [22]. 2-Methyl-1-phenylbut-3-en-1-ol: hexanes/*i*-PrOH = 99:1, 0.5 mL min^−1^, t_R_ (*S,S*; *anti*) = 29.9 min, t_R_ (*S,R*; *syn)* = 32.2 min, t_R_ (*R,R*; *anti*) = 34.2 min, t_R_ (*R,S*; *syn*) = 35.5 min [23].

Ionic liquids and indium metal were purchased from commercial sources and used as received. *N*-Benzylcinchonidium bromide was prepared according to the literature [24]. Allylic bromides and aldehydes were distilled just before use. All reactions were carried out under argon.

### 3.2. General Procedure for the Allylation of Benzaldehyde in Ionic Liquids

A mixture of allyl bromide (138 μL, 1.6 mmol), benzaldehyde (102 μL, 1.0 mmol), and indium (115 mg, 1.0 mmol) was stirred in ionic liquid (1.0 mL) at room temperature for 5 h. Water was added to the reaction mixture and the product was extracted with diethyl ether. The extracts were purified by short column chromatography on silica gel. The yield of 1-phenyl-3-buten-1-ol (**1**) was determined by ^1^H NMR analysis using 1,3,5-trimethylbenzene as the internal standard.

### 3.3. General Procedure for the Pd-Catalyzed Allylation of Benzaldehyde in Ionic Liquids

A mixture of allyl acetate (108 μL, 1.0 mmol), benzaldehyde (51 μL, 0.5 mmol), indium(I) iodide (424 mg, 1.0 mmol), and Pd(PPh_3_)_4_ (29 mg, 0.025 mmol) was stirred in ionic liquid (1.0 mL) at room temperature for 24 h. Dilute hydrochloric acid (1 M) was added to the reaction mixture and the product was extracted with diethyl ether. The organic layer was washed with saturated NaHCO_3_, water, and brine, and dried over Na_2_SO_4_. The yield of 1-phenyl-3-buten-1-ol (**1**) was determined by ^1^H NMR analysis using 1,3,5-trimethylbenzene as the internal standard.

### 3.4. General Procedure for the Crotylation of Benzaldehyde in Ionic Liquids

A mixture of crotyl bromide (81 μL, 0.8 mmol), benzaldehyde (51 μL, 0.50 mmol), and indium (58 mg, 0.50 mmol) was stirred in ionic liquid (1.0 mL) at room temperature for 1 h. Dilute hydrochloric acid (1 M) was added to the reaction mixture and the product was extracted with diethyl ether. The organic layer was washed with saturated NaHCO_3_, water, and brine, and dried over Na_2_SO_4_. The yield of **2** was determined by ^1^H-NMR analysis using 1,3,5-trimethylbenzene as the internal standard.

### 3.5. Crotylation of Benzaldehyde in the Presence of Additives

A mixture of crotyl bromide (81 μL, 0.80 mmol), benzaldehyde (58 μL, 0.50 mmol), indium (58 mg, 0.50 mmol), and additive was stirred in [bmim][BF_4_] (1.0 mL) at room temperature for 1 h. Dilute hydrochloric acid (1 M) was added to the reaction mixture and the product was extracted with diethyl ether. The organic layer was washed with saturated NaHCO_3_, water, and brine, and dried over Na_2_SO_4_. The yield of **2** was determined by ^1^H NMR analysis using 1,3,5-trimethylbenzene as the internal standard.

### 3.6. General Procedure for the Asymmetric Indium-Mediated Allylation of Benzaldehyde Using Cinchonidine

The reaction in entry 10 is described as a representative typical procedure. A mixture of allyl bromide (69 μL, 0.8 mmol), indium (58 mg, 0.50 mmol), and cinchonidine (147 mg, 0.50 mmol) was stirred in [bmim][OTf] (1.0 mL) at room temperature for 3 h. To the resulting mixture a solution of benzaldehyde (25 μL, 0.25 mmol) in CH_2_Cl_2_ (1 mL) was added and the mixture was stirred at −78 °C for another 15 h. The mixture was quenched with dilute hydrochloric acid (1 M, 2 mL) and the product extracted with diethyl ether. The organic layer was washed with saturated NaHCO_3_, water, and brine, and dried over Na_2_SO_4_. The solvent was removed under reduced pressure to give a slightly yellow oil. The yield of 1-phenyl-3-buten-1-ol (**1**) was determined by ^1^H-NMR analysis using 1,3,5-trimethylbenzene as the internal standard (34%), and the ee for (*R*)-**1** was determined by HPLC analysis (61%).

### 3.7. Product Characterization

*1-Phenyl-3-buten-1-ol* (**1**) [22]. ^1^H-NMR (300 MHz, CDCl_3_): δ 2.05 (brs, 1H), 2.47–2.57 (m, 2H), 4.72–4.77 (q, *J* = 7.5 Hz, 1H), 5.12–5.20 (m, 2H), 5.75–5.89 (m, 1H), 7.25–7.37 (m, 5H).

*2-Methyl-1-phenyl-3-buten-1-ol* (**2**) [25]. ^1^H-NMR (300 MHz, CDCl_3_) *syn* isomer: δ 1.01 (d, *J* = 6.6 Hz, 3H), 1.93 (brs, 1H), 2.45–2.62 (m, 1H), 4.62 (d, *J* = 4.8 Hz, 1H), 5.04–5.08 (m, 2H), 5.71–5.88 (m, 1H), 7.26–7.337 (m, 5H); *anti* isomer: δ 0.87 (d, *J* = 6.9 Hz, 3H), 1.93 (bs, 1H), 4.36 (d, *J* = 5.2 Hz, 1H), 5.17–5.24 (m, 1H).

*1-Phenyl-3-penten-1-ol* (**3**) [18]. ^1^H-NMR (300 MHz, CDCl_3_) *(E)-* isomer: δ 1.61 (dd, *J* = 6.8, 0.8 Hz, 3H), 2.00 (s, 1H), 2.34–2.63 (m, 2H), 4.66–4.75 (m, 1H), 5.38–5.47 (m, 1H), 5.55–5.68 (m, 1H), 7.26–7.39 (m, 5H); (*Z)-* isomer: δ 1.69 (dd, *J* = 6.2 Hz, 0.9 Hz, 3H), 2.06 (s, 1H).

*2-Methyl-1-(2-pyridinyl)-3-buten-1-ol* (**4**) [20].^1^H-NMR (300 MHz, CDCl_3_) *syn* isomer: δ 0.91 (d, *J* = 6.9 Hz, 3H), 2.57–2.73 (m, 1H), 4.12 (t, *J* = 6.3 Hz, 1H), 4.71 (t, *J* = 4.8 Hz, 1H), 5.00–5.10 (m, 2H), 5.86–5.99 (m, 1H), 7.18–7.26 (m, 2H), 7.67 (t, *J* = 7.8 Hz, 1H), 8.55 (d, *J* = 4.8 Hz, 1H); *anti* isomer: δ 1.08 (d, *J* = 6.9 Hz, 3H), 4.64 (t, *J* = 4.8 Hz, 1H), 5.69–5.81 (m, 1H).

*2-Methyl-1-(3-pyridinyl)-3-buten-1-ol* (**5**) [26]. ^1^H-NMR (300 MHz, CDCl_3_) *syn* isomer: δ 0.91 (d, *J* = 6.9 Hz, 3H), 2.48 (sext, *J* = 7.5 Hz, 1H), 4.43 (d, *J* = 7.5 Hz, 1H), 5.03–5.11 (m, 1H), 5.18–5.23 (m, 1H), 5.70–5.85 (m, 1H), 7.27–7.30 (m, 1H), 7.65–7.71 (m, 1H), 8.53 (brs, 2H); *anti* isomer: δ 1.02 (d, *J* = 6.6 Hz, 3H), 2.60 (sext, *J* = 6.6 Hz, 1H), 4.67 (d, *J* = 5.4 Hz, 1H).

## 4. Conclusions

The choice of ionic liquid is important to achieve a clean In-mediated allylation and crotylation of carbonyl compounds. From a screening of ionic liquids, [bmim][BF_4_] was found to be the most promising solvent for both the In-promoted couplings of allyl bromide to carbonyl compounds and the Pd-catalyzed In(I)-mediated version. Instead, when crotyl bromide is employed, the use of pure [bmim][BF_4_] has detrimental effects, and the addition of appropriate halide salts or amines is crucial for a successful crotylation leading to the γ-adduct. Instead, the addition of alcohols and water in [bmim][BF_4_] favor the formation of the α-adduct isomer. Furthermore, cinchonidine functions as a chiral promoter in the coupling of allyl bromide and benzaldehyde in [bmim][BF_4_]/CH_2_Cl_2_, affording the corresponding homoallylic alcohol with up to 62% ee.

## Figures and Tables

**Table 1:**
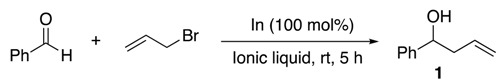
Indium-mediated allylation in ionic liquid. ^a^

Entry	Ionic Liquid	Yield of 1 (%) ^b^
1	[bmim][BF_4_]	95
2	[bpy][BF_4_]	82
3	[bmim][(CN)_2_N]	88
4	[bmim][OTf]	69
5 ^c^	[bmim][Cl]	41
6	[bmim][NTf_2_]	0

^a^ All reactions were carried out with allyl bromide (1.6 mmol), benzaldehyde (1.0 mmol) and indium (1.0 mmol) in ionic liquid (1.0 mL) at room temperature for 5 h; ^b^ NMR yields determined using 1,3,5-trimethoxybenzene as the internal standard; ^c^ Reaction carried out at 73 °C.

**Table 2:**
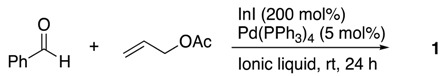
Pd-catalyzed indium-mediated allylation in ionic liquid ^a^.

Entry	Ionic Liquid	Yield of 1 (%) ^b^
1	[bmim][BF_4_]	75
2	[bpy][BF_4_]	61
3	[bmim][OTf]	50
4	[bmim][(CN)_2_N]	16

^a^ All reactions were carried out with allyl acetate (1.0 mmol), benzaldehyde (0.50 mmol) and indium(I) iodide (1.0 mmol), and Pd(PPh_3_)_4_ (0.025 mmol) in ionic liquid (1.0 mL) at room temperature for 24 h; ^b^ NMR yields determined using 1,3,5-trimethoxybenzene as the internal standard.

**Table 3:**
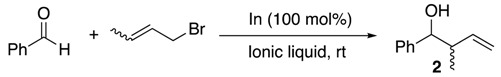
Crotylation of benzaldehyde in ionic liquid ^a^.

Entry	Ionic Liquid	Time (h)	Yield of 2 (%) ^b^
1	[bmim][(CN)_2_N]	0.5	80 (58:42) ^c^
2	[bmim][OTf]	1	67 (55:45) ^c^
3	[bmim][BF_4_]	1	Complex mixture

^a^ All reactions were carried out at a 0.5 mmol scale of benzaldehyde at room temperature in ionic liquid (1 mL); ^b^ Determined by ^1^H-NMR using 1,3,5-trimethoxybenzene as the internal standard; ^c^ *Syn*:*anti*.

**Table 4:**
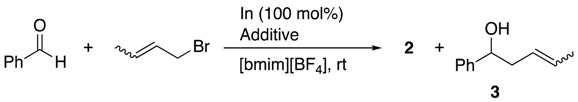
Crotylation of benzaldehyde in the presence of additives ^a^.

Entry	Additive (mol %)	Time (h)	NMR Yield (%) (*syn*:*anti*) ^b^
1	[bmim][Cl] (114)	1	**2**: 90 (54:46)
2	LiCl (100)	1	**2**: 92 (59:41)
3 ^c^	LiCl (10)	1	**2**: 8 (66:34) ^d^
4	LiCl (200)	1	**2**: 58 (57:43)
5	[bmim][Cl] (10)	5	**2**: 65 (58:42)
6	[bmim][Br] (10)	5	**2**: 80 (63:37)
7	LiBr (10)	1	**2**: 62 (63:37)
8	PPh_3_ (100)	2	**2**: 78 (56:44)
9	1-Butylamine (100)	3	**2**: 48 (58:42)
10	TMEDA ^e^ (100)	18	trace
11	Pyridine (100)	6	**2**: 75 (57:43)
12	Cinchonidine (100)	6	**2**: 82 (54:46)
13	1-Hexanol (100)	6	**2**: 26 (73:27) ^d^, **3**: 41 (47:53) ^d,f^
14	Phenol (100)	6	0
15	Diethyl l-tartrate (100)	3	**2**: 19 (75:25) ^d^, **3**: 33 (55:45) ^d,f^
16	H_2_O (100)	6	**2**: 45 (74:26) ^d^, **3**: 42 (48:52) ^d,f^

^a^ All reactions were carried out at a 0.5 mmol scale of benzaldehyde at room temperature in [bmim][BF_4_] (1 mL); ^b^ 1,3,5-Trimethoxybenzene was used as the internal standard; ^c^ With 1.0 mmol scale; ^d^ Isolated yield; ^e^ Tetramethylethylenediamine; ^f^ *E*:*Z*.

**Table 5:**
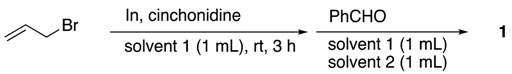
Asymmetric indium-mediated allylation of benzaldehyde using cinchonidine ^a^.

Entry	Solvent Solvent 1/Solvent 2	Temp. (°C)	Time (h)	Yield (%)	Ee (%) ^b^
1	[bmim][BF_4_]	rt	15	50	2
2	[bmim][BF_4_]/CH_2_Cl_2_	rt	15	90	33
3	[bmim][BF_4_]/CH_2_Cl_2_	−78	15	41	2
4	[bmim][BF_4_]/CH_2_Cl_2_	40	15	51	20
5 ^c^	[bmim][BF_4_]/CH_2_Cl_2_	rt	1	81	6
6	[bpy][BF_4_]/CH_2_Cl_2_	rt	15	71	28
7	[bpy][BF_4_]/CH_2_Cl_2_	−60	65	76	3
8	[bmim][OTf]	rt	15	81	0
9	[bmim][OTf]/CH_2_Cl_2_	rt	1	98	51
10	[bmim][OTf]/CH_2_Cl_2_	−20	15	23	62
11	[bmim][OTf]/CH_2_Cl_2_	−78	15	34	61

^a^ Benzaldehyde (0.25 mmol), allyl bromide (320 mol %), indium (200 mol %), and cinchonidine (200 mol %) were used; ^b^ Determined by HPLC; ^c^ *N*-Benzylcinchonidium bromide was used instead of cinchonidine.

## Supplementary Materials

The following are available online. ^1^H-NMR of compounds **1**–**5**.
