# Supplementary material for: Indium-Mediated Allylation of Carbonyl Compounds in Ionic Liquids: Effect of Salts in Ionic Liquids
Source: Molecules. 2018 Jul 11;23(7):1696. doi: 10.3390/molecules23071696 (PMC6100023; doi:10.3390/molecules23071696)

Supporting Information

Indium-mediated Allylation of Carbonyl Compounds in Ionic Liquids. Effect of Salts in Ionic Liquids

Tsunehisa Hirashita*, Fusako Takahashi, Takayuki Noda, Yuji Takagi and Shuki Araki

Life Science and Applied Chemistry, Graduate School of Engineering, Nagoya Institute of Technology, Gokiso-cho, Showa-ku, Nagoya 466-8555, Japan;

Table of Contents

1. NMR Spectra of **1** (entry 1, Table 1) S2

2. NMR Spectra of **2** (entry 2, Table 4) S3

3. NMR Spectra of **3** (entry 15, Table 4) S4

4. NMR Spectra of **4** (Scheme 2) S4

5. NMR Spectra of **5** (Scheme 2) S5

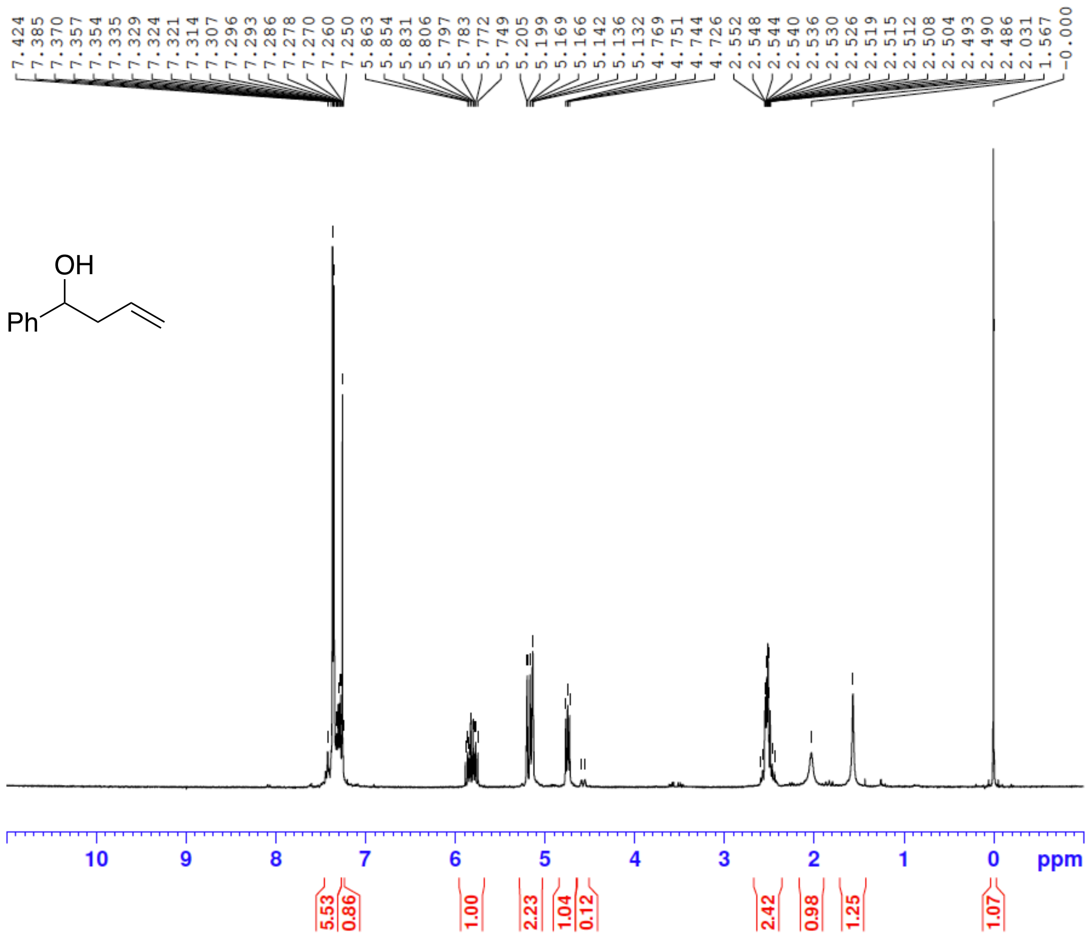

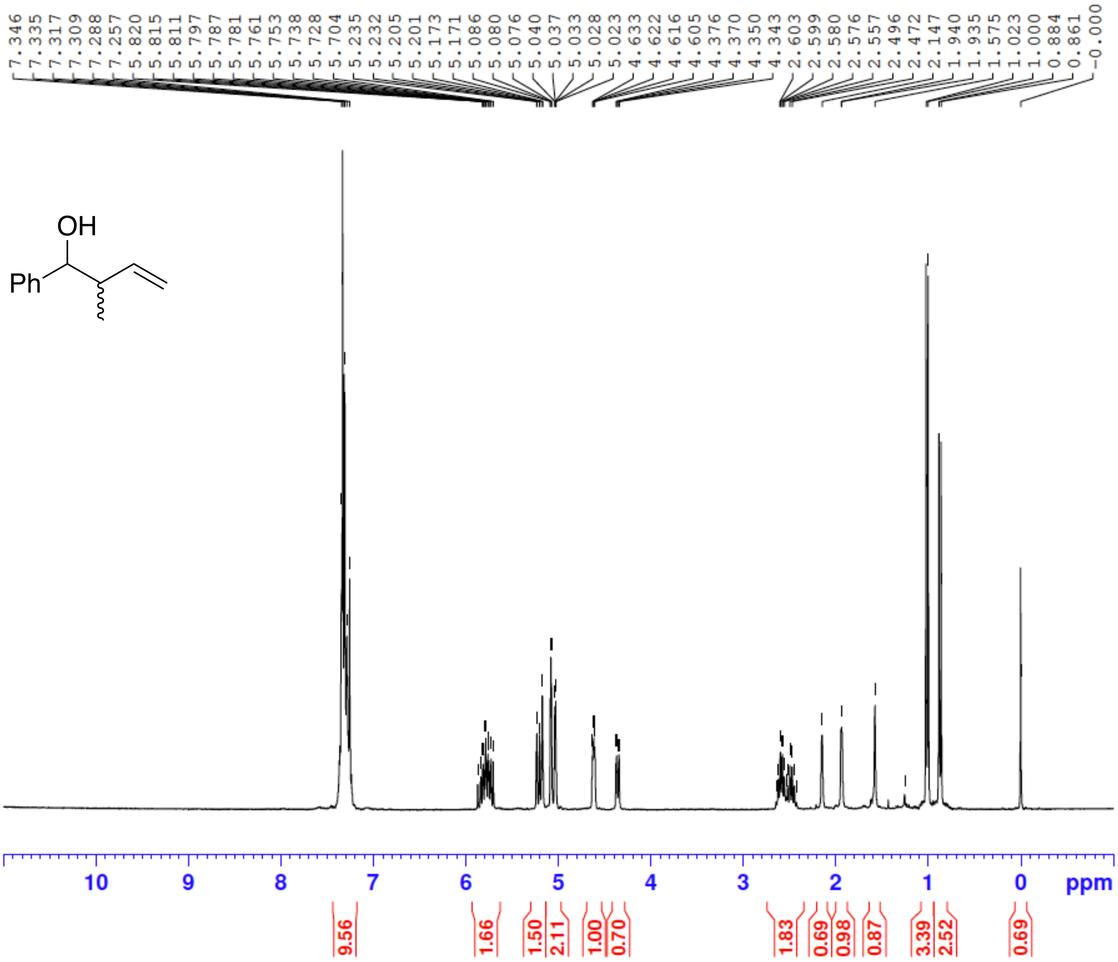

OH
Ph
7.346 7.335 7.317 7.309 7.288 7.257 5.820 5.815 5.811 5.797 5.787 5.781 5.761 5.753 5.738 5.728 5.704 5.235 5.232 5.205 5.201 5.173 5.171 5.086 5.080 5.076 5.040 5.037 5.033 5.028 5.023 4.633 4.622 4.616 4.605 4.376 4.370 4.350 4.343 2.603 2.599 2.580 2.576 2.557 2.496 2.472 2.147 1.940 1.935 1.575 1.023 1.000 0.884 0.861 -0.000
10 9 8 7 6 5 4 3 2 1 0 ppm
9.56 1.66 1.50 2.11 1.00 0.70 1.83 0.69 0.98 0.87 3.39 2.52 0.69

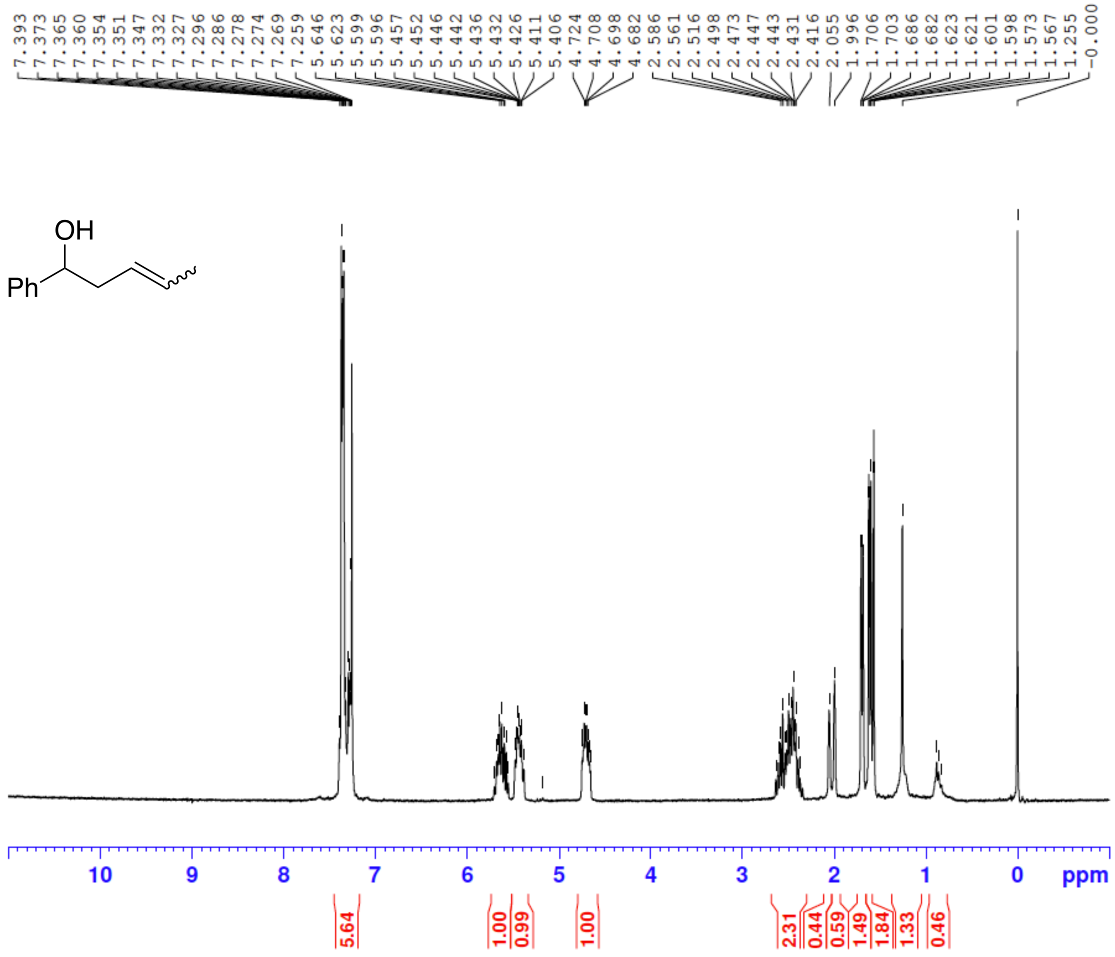

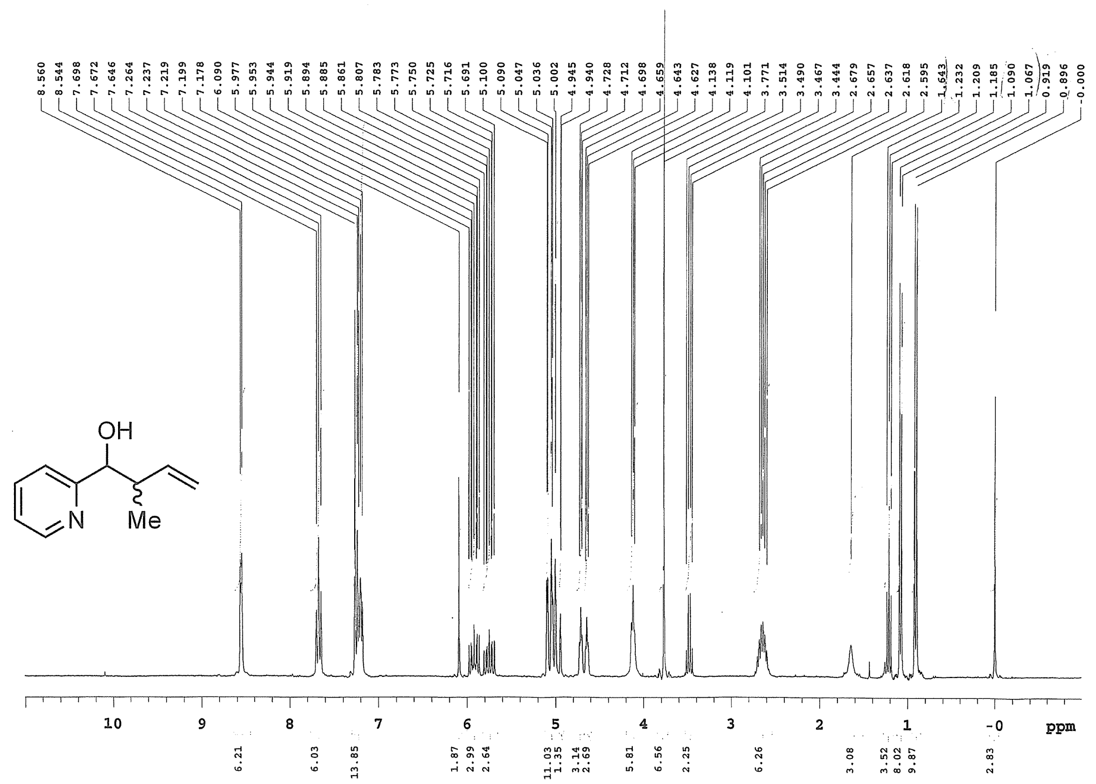

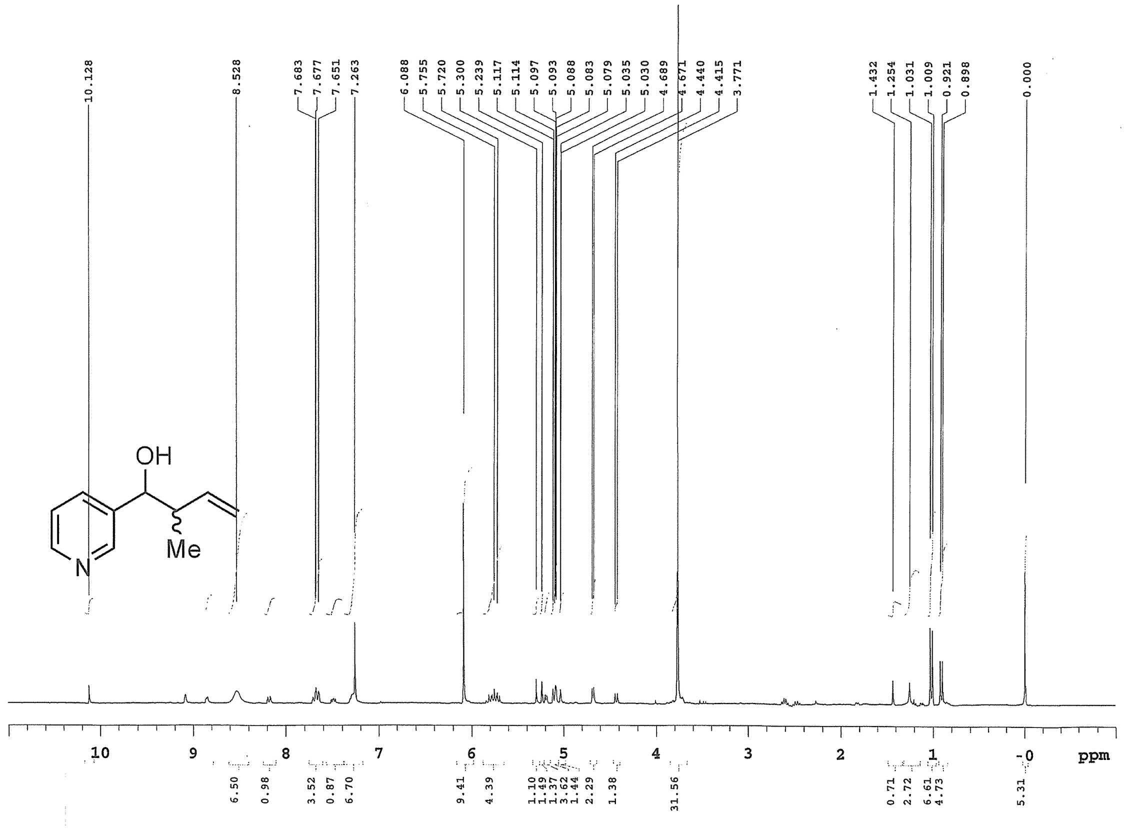

Supplement: Supplementary file 1 [file molecules-23-01696-s001.pdf]
